# The influence of spaceflight on the astronaut salivary microbiome and the search for a microbiome biomarker for viral reactivation

**DOI:** 10.1186/s40168-020-00830-z

**Published:** 2020-04-20

**Authors:** Camilla Urbaniak, Hernan Lorenzi, James Thissen, Crystal Jaing, Brian Crucian, Clarence Sams, Duane Pierson, Kasthuri Venkateswaran, Satish Mehta

**Affiliations:** 1 NASA Jet Propulsion Laboratory, California Institute of Technology, Pasadena, CA USA; 2 Department of Infectious Diseases, J. Craig Venter Institute, Rockville, MD USA; 3 Lawrence Livermore National Laboratory, Livermore, CA USA; 4 NASA Johnson Space Center, Houston, TX USA; 5 JES Tech, NASA Johnson Space Center, Houston, TX USA

**Keywords:** Salivary microbiome, Viral reactivation, Astronaut microbiome, Spaceflight, International space station

## Abstract

**Background:**

Spaceflight impacts astronauts in many ways but little is known on how spaceflight affects the salivary microbiome and the consequences of these changes on astronaut health, such as viral reactivation. In order to understand this, the salivary microbiome was analyzed with 16S rRNA gene amplicon sequencing, and saliva viral titers were analyzed with quantitative polymerase chain reaction (qPCR) with primers specific for Epstein-Barr virus (EBV), herpes simplex virus (HSV), and varicella zoster virus (VZV) from 10 astronauts pre-flight, in-flight, and post-flight.

**Results:**

*Streptococcus* was the most abundant organism in the saliva, making up 8% of the total organisms detected, and their diversity decreased during spaceflight. Other organisms that had statistically significant changes were *Proteobacteria* and *Fusobacteria* which increased during flight and *Actinobacteria* which decreased during flight. At the genus level, *Catonella*, *Megasphera*, and *Actinobacillus* were absent in more than half of saliva samples collected pre-flight but were then detected during flight. In those subjects that already had these genera pre-flight, their relative abundances increased during flight. Correlation analyses between the microbiome and viral titers revealed a positive correlation with *Gracilibacteria*, *Absconditabacteria*, and *Abiotrophia* and a negative correlation between *Oribacterium*, *Veillonella*, and *Haemophilus*. There was also a significant positive correlation between microbiome richness and EBV viral titers.

**Conclusions:**

This is the first study to look at how the salivary microbiome changes as a result of spaceflight and the search for bacterial biomarkers for viral reactivation. Further studies examining the role of specific organisms that were shown to be correlative and predictive in viral reactivation, a serious problem in astronauts during spaceflight, could lead to mitigation strategies to help prevent disease during both short and long duration space missions.

Video abstract.

## Introduction

The World Health Organization published a surveillance strategy report in 2015 stating that worldwide, non-infectious disease (NIDs) represented 43% of global disease burden and was expected to be responsible for 60% of the disease burden and 73% of all deaths by 2020s [1]. In 2016, 80% of NID deaths were due to cancers, cardiovascular diseases, chronic respiratory diseases, and diabetes, with the remaining 20% due to immune disorders, digestive issues, genitourinary disease, and oral or dental conditions [2]. The collection of bacteria and less dominant micro-organisms that inhabit our body is termed the human microbiome and plays an integral role in maintaining health. Changes in the composition of one’s microbiome may promote the development of the aforementioned NIDs, as individuals with inflammatory bowel disease [3, 4], asthma [5], diabetes [6], cardiovascular disease [7], colorectal cancer [8], and breast cancer [9] have different bacterial communities than healthy individuals. While it is still unclear whether these microbial differences are a consequence or a cause of disease, there is evidence in favor of the latter, as healthy animals transplanted with feces from those with obesity [10], colitis [11], or colorectal cancer [12] then go on to develop disease.

The importance of the human microbiome in health promotion is just as relevant to astronauts and perhaps even more so, as medical procedures and facilities are limited during spaceflight. As of 2018, there have been 557 astronauts/cosmonauts that have flown to outer space with well-documented physiological and immunological issues, such as bone loss, muscle atrophy, fatigue, elevated cortisol levels, nausea, skin/urinary tract/upper-respiratory tract infections, and impaired innate/adaptive immunity [13–15]. However, data is still limited on how the human microbiome, which plays an integral role in human health, is impacted by spaceflight and whether changes in the astronaut microbiome contribute to the conditions experienced by many astronauts.

Culture analyses conducted during the Skylab missions suggest that oral microbial populations may change during spaceflight, as increased counts of anaerobic bacteria from intra-oral sites were recorded in-flight compared to pre-flight samples from 18 astronauts studied [16]. The effects of spaceflight on the oral microbiome should be examined in detail as imbalances have been implicated in oral cancer, caries, periodontitis, immune regulation, cardiovascular disease, and diabetes [17–21], and thus could significantly impact the health of astronauts during long-term spaceflight (i.e., manned missions to Mars).

Reactivation of latent herpesviruses has been documented in astronauts for years. Reactivation of Epstein-Barr virus (EBV), varicella-zoster virus (VZV), and cytomegalovirus (CMV) have been observed during both short (10–16 days) [22] and long (60–180 days) [23] duration ISS missions with longer missions producing higher viral titers and prolonged shedding upon return to Earth (shedding for 30 days post-flight [long duration mission] vs shedding for 5 days post flight [shorter duration]) [23]. The impact of EBV shedding, for example, on crew health could be limited to the minor symptoms of infection to an increased risk of lymphoma, oral squamous cell carcinoma, and mononucleosis [24, 25]. The role of the oral microbiome in viral reactivation during spaceflight has not yet been studied, but may be a key factor in this phenomenon. Its role may be indirect, through immune modulation or directly, through bacterial-viral interactions. Studies have shown that short-chain fatty acids produced by oral bacteria induce reactivation of latent human immunodeficiency virus (HIV), Kaposi’s sarcoma herpesvirus (KSHV), and EBV, by activating viral promotors and/or causing epigenetic modifications in the inserted viral genome [26–29].

The aim of this study was to examine, for the first time, whether and how the salivary microbiome changes as a result of spaceflight and whether there is a microbiome signature that may be influential in preventing or promoting viral reactivation in humans. The data acquired from this study could help inform the NASA medical team about the role of bacterial signatures in maintaining crew health while in space.

## Results

Eighty-nine samples from ten astronauts were analyzed by 16S rRNA gene sequencing for microbiome analysis and qPCR for viral titer measurements. The timepoints for each sample collected the viral status, and flight history are presented in Dataset S1.

### Impact of spaceflight on the salivary microbiome

**Fig. 1:**
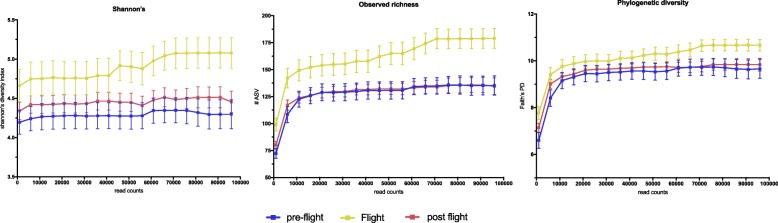
Alpha diversity of salivary microbiome. Saliva samples were collected from 10 astronauts at various time points before flying to the ISS (pre-flight), while on the ISS (Flight) and upon return to Earth (post-flight). Diversity was measured using Shannon’s diversity index (left panel) and Faith’s phylogenetic diversity (right panel). Richness was measured by counting the number of unique amplicon sequence variants (ASV) within each sample (middle panel). Reads were rarified to different counts (x-axis) and the values for each rarefied read count plotted (y-axis). For all 3 metrics tested, alpha diversity was the highest in-flight, with post-flight samples decreasing to pre-flight values. Kruskal-Wallis one-way analysis of variance followed by the Benjamini-Hochberg multiple comparison post-hoc test (Significance threshold *P* < 0.05).

Alpha diversity of the salivary microbiome was assessed with Shannon’s diversity (measure of richness and evenness), Faith’s phylogenetic diversity (phylogenetic difference between the observed sequences), and community richness (number. of observed amplicon sequence variants [ASVs]). All three metrics showed that microbiome diversity and richness increased during spaceflight (*P* < 0.05) but then dropped back to pre-flight levels upon return to Earth (Fig. 1).

*Streptococcus* is the most abundant organism in the saliva, and in this dataset consisted of 58 amplicon sequence variants, making up 8% of the total. For this reason, it was of interest to examine how alpha diversity of *Streptococcus* sequences changed as a result of spaceflight. Unlike the collective microbiome, Shannon’s diversity of *Streptococcus* was lower in spaceflight samples compared to pre-flight and post-flight samples (*P* < 0.05), and while diversity did rebound after flight, it did not reach pre-flight values (Figure S1A). On the other hand, while pre-flight and in-flight samples did not show significant differences in richness (though the number of observed *Streptococcus* sequences did decrease slightly in-flight), there was a significant decrease in the number of streptococcal sequences in samples collected post-flight (Figure S1B).

**Fig. 2:**
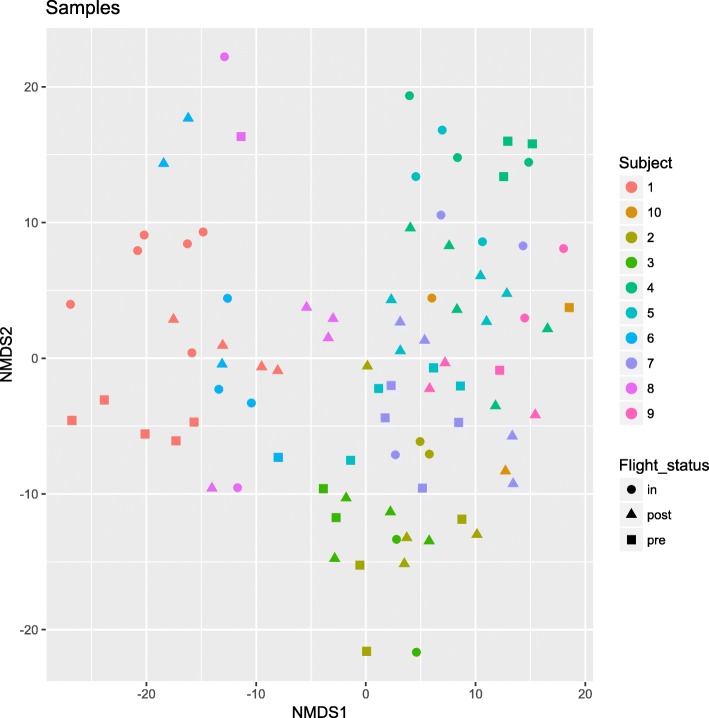
Beta diversity of the salivary microbiome. Non-metric multidimensional scaling (NMDS) ordination of the salivary microbiome collected from ten astronauts collected pre-flight, in-flight (i.e., on the ISS), and post-flight. Each dot on the graph represents a sample, with the different colors representing a subject, and the different shapes representing a flight status. The closer the dots are to each other on the graph, the more similar the samples are in microbiome composition. The plots represent ASV sequences summarized, based on taxonomy, to the genus level and then clr transformed. No distinct clusters were observed, indicating no overall microbiome difference between subjects and based on flight status. The same analysis was performed at the ASV level and showed the same trends as at the genus level. The data used for analysis was clr transformed and Euclidean distances used.

**Fig. 3:**
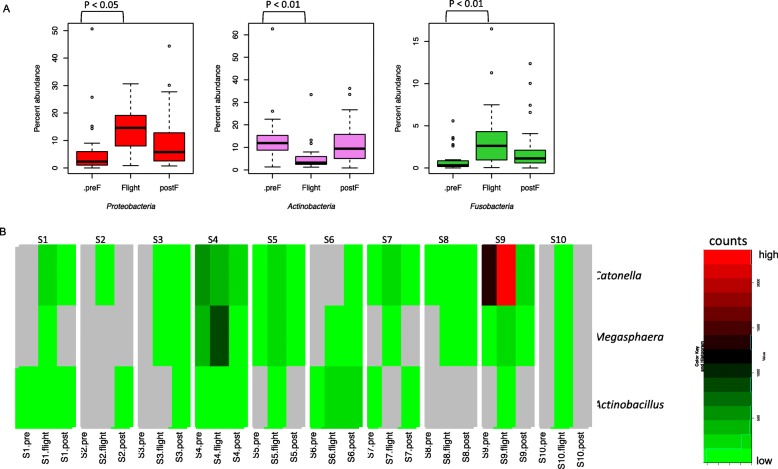
Differential abundance of bacteria based on flight status. **a** ALDEx2 statistically analysis was performed on sequences summarized to the phylum level. Significance was based on the Benjamini-Hochberg corrected *p* value of the Wilcoxon rank test (significance threshold *P* < 0.05). Out of the nine phyla detected in saliva, three showed statistically significant differences as a result of spaceflight. The changes in their relative abundances between pre-flight, in-flight, and post-flight are shown with boxplots. The box in each graph signifies the 75% (upper) and 25% (lower) quartiles and thus shows the percent abundances for 50% of the samples. The black line inside the box represents the median. The bottom whisker represents the lowest datum still within the 1.5 interquartile range (IQR) of the lower quartile, with the top whisker representing the highest datum still within the 1.5 IQR of the upper quartile. Open circles are outliers. **b** In half of the subjects tested, three genera, *Catonella*, *Megasphaera*, and *Actinobacillus* were not present in pre-flight samples, but were detected in samples collected during flight. In those that already had these genera pre-flight, their relative abundances increased in-flight. The read counts for these three organisms in all ten subjects are shown in a heatmap. Gray indicates that no reads were detected while a gradient from green to red shows low counts and high counts, respectively

Beta diversity of the salivary microbiome was also compared. Non-metric multidimensional scaling (NMDS) of the centered log ratio (clr) transformed data did not show any distinct clustering, indicating no overall microbiome differences between subjects and based on flight status (Fig. 2). Statistical analyses using ALDEx2 did, however, show that the relative abundances of three phyla changed over time, with *Proteobacteria* and *Fusobacteria* increasing in relative abundances during flight and *Actinobacteria* decreasing (Fig. 3a). While post-flight samples resembled pre-flight values more so than flight values, they were either higher (*Proteobacteria* and *Fusobacteria*) or lower (*Actinobacteria*) than what was observed pre-flight. At the genus level, in more than half of the subjects tested, *Catonella*, *Megasphera*, and *Actinobacillus* were absent in saliva collected pre-flight but were then detected during flight (Fig. 3b**)**. In those subjects that already had these genera pre-flight, their relative abundances increased during flight (Fig. 3b). The read counts of all genera detected and grouped by subject, and flight status is summarized as a heatmap in Figure S2.

**Fig. 4:**
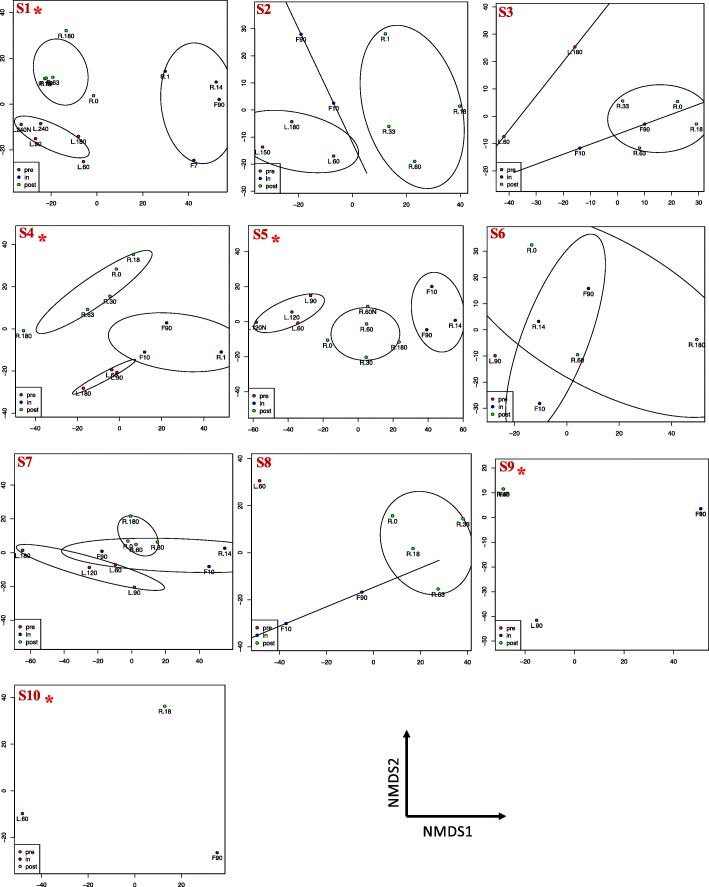
Comparison of salivary microbiome based on flight status at the individual level. NMDS ordination, with Euclidean distances, was generated at the ASV level of centered log ratio (clr) data for each individual. Each dot on the graph represents a sample, with the different colors representing whether the sample was collected pre-flight (red), in-flight (blue), or post flight (green). The closer the dots are to each other on the graph, the more similar the samples are in microbiome composition. The subjects are labeled in the top left end corner of each box and an asterisk (*) beside the subject number indicates microbiome differences based on flight status. Differences are based on ellipses drawn around samples based on a 99% confidence interval. Samples that belong to the same flight status and in the same ellipse, and which do not overlap with other ellipses, are considered distinct groups, thereby having different microbiome profiles

While NMDS analysis of the salivary microbiome showed no differences based on flight status at the population level (Fig. 2), studies have shown that in some cases, population level analyses may mask microbiome differences that occur at the individual level [30]. For this reason, NMDS ordination was performed for each subject, in order to better understand the impact of spaceflight on the salivary microbiome. Five out of the 10 subjects clustered based on flight status with pre-flight, flight, and post-flight samples all different from each other (Fig. 4). It is important to note that different taxa were responsible for the differences based on flight status in these five individuals. For the other five subjects, while there was some overlap between samples collected from the different flight groups, pre-flight samples were never similar to post-flight samples.

### Association between viral reactivation and salivary microbiome

**Fig. 5:**
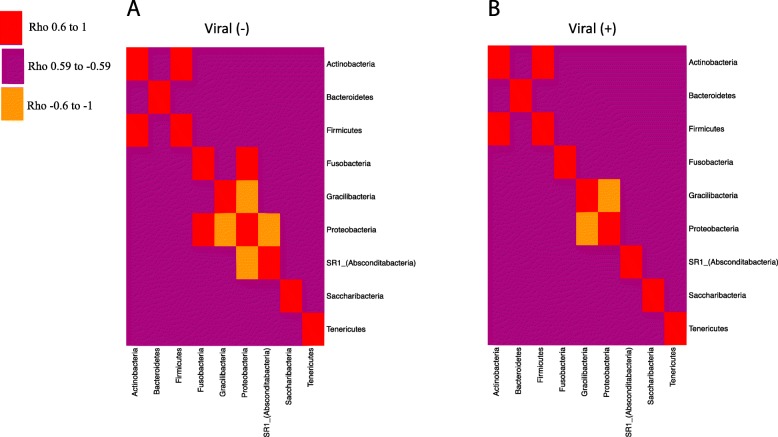
Microbiome correlations based on viral status. 16S rRNA sequences were summarized to the phylum level, and a Spearman’s correlation analysis was performed to assess correlations between the bacterial communities in the saliva. Two separate correlation tests were performed, one with samples that were negative for either EBV or HSV-1 (as detected by qPCR) (**a**) and the second, using samples that were positive for virus (based on qPCR) (**b**). The heatmaps display the Spearman’s rho values, with red boxes indicating rho values above 0.6 (strong positive correlation) and yellow values indicating rho values less than − 0.6 (strong inverse correlation). The maroon boxes represent no correlation. All red and yellow microbiome correlations had Benjamini-Hochberg corrected *p* values of < 0.05. As observed from these heatmaps, there are more bacterial correlations in saliva that are devoid of EBV and HSV-1 compared to saliva with viral shedding

The correlation between members of the salivary microbiome was examined for saliva samples that were either positive or negative for EBV or HSV-1 (as determined by qPCR). Associations between phyla changed depending on whether samples were negative or positive for virus. In saliva devoid of virus (no viral reactivation), positive correlations were present between (i) *Actinobacteria* and *Firmicutes* and (ii) *Fusobacteria* and *Proteobacteria*, with an inverse relation between (i) *Gracilibacteria* and *Proteobacteria* and (ii) *Absconditabacteria* and *Proteobacteria* (Fig. 5a). However, in saliva with viral reactivation, all bacterial correlations disappeared except for the inverse association between *Gracilibacteria* and *Proteobacteria* (Fig. 5b).

**Fig. 6:**
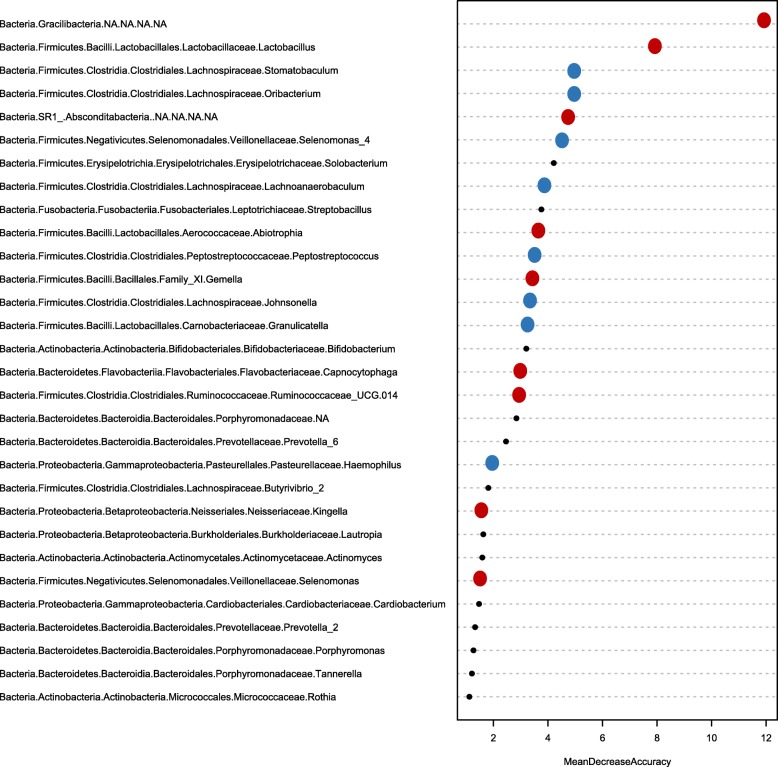
Salivary microbiome can predict viral status. Results from the random forest classifier showing the bacterial genera that are the most discriminatory between viral positive (qPCR detection of EBV or HSC-1) and viral negative samples, in descending order. OOB error rate was 24%, and LOOCV accuracy was 75%. Taxa are colored based on whether they were significantly higher in viral positive samples (red), viral negative samples (blue), or not (black) based on the Wilcoxon rank test with a Benjamini-Hochberg correction for multiple comparisons. Significance *P* < 0.05

To determine which genera were most discriminatory between viral negative and viral positive samples, the machine-learning algorithm random forests (RF) was applied to genus level abundance data. The out-of-bag (OOB) error rate was 24%, and the leave-one-out cross validation (LOOCV) showed our model to be 75% accurate. Taxa that proved most discriminatory between the two groups were ordered according to mean decrease in accuracy (Fig. 6). *Gracilibacteria* was the most predictive followed by *Lactobacillus*, *Stomatobaculum*, and *Oribacetrium*. RF was also applied to *Streptococcus* sequences due to their abundance in the samples. With an OOB error rate of 19% and a LOOCV accuracy of 81%, *Streptococcus* strains were discriminatory between the cohorts with *S. mutans* being the most predictive of viral status (Fig S3).

**Fig. 7:**
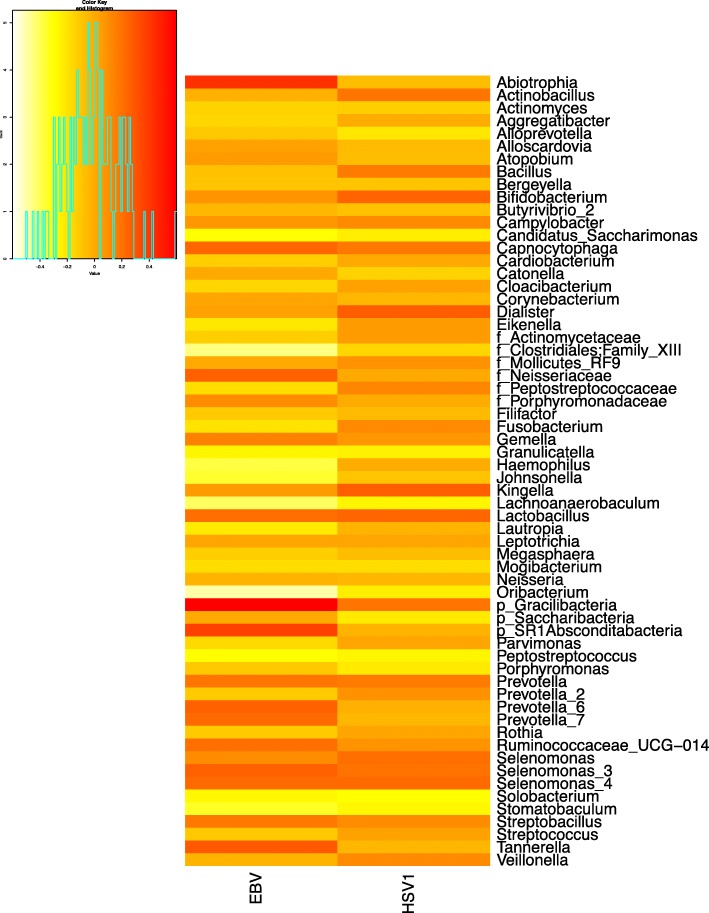
Correlation between salivary microbiome and viral load. 16S rRNA sequences were summarized to the genus level, and a Spearman’s correlation analysis was performed between qPCR viral copy number and relative abundances of bacteria. The heatmap presented shows the Spearman’s rho values, with red representing the highest positive values (positive correlation) and light yellow representing the lowest negative values (inverse correlation). Of those that did not resolve to the genus level, but only to the family or phylum level, this is identified on the heatmap as “f_” for family level resolution and “p_” for phylum level resolution. A summary of all rho values and their associated Benjamini-Hochberg corrected *p* values are listed in Dataset S2

Correlations were examined between viral copy numbers (determined by qPCR) and the relative abundances of the microbiome (16S rRNA sequencing). Spearman’s rho correlation values between EBV or HSV-1 viral abundance and relative abundances of the salivary microbiome are shown in the heatmap in Fig. 7. For EBV, the strongest positive correlation was observed for the candidate phyla *Gracilibacteria* and *Absconditabacteria* and the genus *Abiotrophia*. The strongest inverse correlation was observed for *Oribacterium*, *Clostridiales_Family*XIII, *Lachnoanaerobaculum*, *Haemophilus*, *Johnsonella*, and *Stomatobaculum*. No significant correlations were, however, observed between HSV-1 viral abundance and microbiome relative abundances, as all Spearman’s rho correlations were under +/− 0.3 (“weak correlations”), and none of the correlations had *p* values less than 0.05. A summary of Spearman’s rho and *p* values for all genera for each virus is summarized in Dataset S2.

Correlations were next examined between viral abundance and microbiome diversity and richness. No significant correlations between qPCR viral load and Shannon’s diversity index was observed for EBV (*p* value 0.35, tau = − 0.03) nor HSV-1 (*p* value = 0.87, tau = − 0.092). However, when microbiome richness was examined, there was a significant positive correlation for EBV (*p* value = 0.005, tau = 0.25) but not for HSV-1 (*p* value = 0.34, tau = 0.09).

## Discussion

### Viral reactivation

The human microbiome has become a powerful tool to help predict and modulate health and disease. The oral microbiome specifically has been implicated in modulating oral cancer, caries, periodontitis, immunity, cardiovascular disease, and diabetes [17–21]. However, there have been limited studies to date that have explored the possible interplay between the oral microbiome and latent viral reactivation and propagation [26–29]. One of the aims of this study was thus to determine whether there was a salivary microbiome signature that could help predict whether an astronaut would be more prone or better protected against EBV, HSV-1, or VZV reactivation. As none of the 89 saliva samples collected were positive for VZV, the analyses presented in this paper are based on EBV (30% of samples positive) and HSV-1 (22% of samples positive).

A positive correlation was found between EBV copy number (qPCR viral data) and bacterial richness (an alpha diversity measure of 16S rRNA observed sequences) which suggests that as the number of bacterial strains increase within a sample, the viral load also increases. In relation to health and disease, high bacterial richness in the saliva has been associated with poor oral health (decayed teeth, periodontitis, and poor oral hygiene) [31, 32], and in the vagina, high bacterial diversity has been linked to bacterial vaginosis [33]. It is unknown how increased bacterial richness may promote EBV reactivation and/or replication, but one possibility is that certain strains, that may be beneficial in maintaining latency and/or curbing viral growth, become diluted in the sample (i.e., relative abundance decreases) and thus do not have as profound an impact on the host. Another hypothesis could be that the additional strains not present in virus negative samples produce metabolites that (i) induce reactivation, (ii) directly interact with the viral particle to increase propagation, and/or (iii) minimize the ability of the host to properly clear viral particles. To note, there is the possibility that weakening of the astronauts’ immune response during spaceflight facilitates EBV reactivation and the growth of bacterial strains that otherwise would be controlled by the immune response, remaining undetected. While there was a correlation between EBV and the microbiome, none existed for HSV-1. Even though EBV and HSV-1 are both members of the herpesvirus family (*Herpesviridae*), the microbiome-viral interactions may differ and thus varied responses will ensue.

The Spearman’s correlation analysis showed an inverse correlation between EBV copy number and the relative abundances of the genera *Oribacterium*, *Lachnoanaerobaculum*, *Haemophilus*, *Johnsonella*, and *Stomatobaculum*, which, with the exception of *Haemophilus*, all belong to the *Lachnospiraceae* family. These genera could also accurately predict, as indicated by random forests, whether a saliva sample was positive or negative for viral titers. Since this study showed that a decrease in the relative abundances of these bacteria are associated with higher viral titers, it suggests a protective role of these bacteria in maintaining viral latency and/or inhibiting viral growth once the lytic cycle is switched on.

Members of the genus *Oribacterium* are non-spore forming, gram positive, motile, and strict anaerobes that have been isolated from human subgingival plaque [34]. There are very few studies that make reference to it in terms of health and disease, but there is one record of *Oribacterium* being enriched in saliva samples from healthy control subjects compared to saliva from patients with oral squamous cell carcinoma [35]. The major fermentation end product of two of the three species in this genus, which were both found in our dataset, *O. parvum* and *O. sinus*, is lactate [34, 36]. Lactate levels in the saliva could control viral titers once reactivation occurs, as physiological levels of lactic acid present in the vaginal tract have shown potency in HIV and *Chlamydia* inactivation [37, 38].

Lactate also significantly increases the production of hydrogen sulfide (H_2_S) by *Veillonella* [39, 40]. H_2_S has been shown to be protective against pathogenic RNA viruses by decreasing the expression of viral proteins and mRNA during the early stages of replication, [41, 42] though no studies have been published regarding its protective capacity against DNA viruses. *Veillonella*, one of the major H_2_S producers in the oral cavity [43], had a significantly (*p* < 0.05) higher relative abundance in samples that came from subjects that never had a reactivation episode compared to samples that were negative for virus but came from subjects that had a reactivation episode at least once (Figure S4A). It was also the most predictive organism, as assessed by random forests, for these groups (Figure S4C). This raises the question as to whether *Veillonella* or specifically H_2_S, which is also produced by all three *Lachnoanaerobaculum* species [44], could play a role in maintaining viral latency. Hydrogen peroxide (H_2_O_2_) is a potent inducer of latent viral proteins and shown to induce reactivation of EBV [45], Karposi’s sarcoma virus [46], and HIV [47]. In a study involving wild-type and H_2_S deficient strains of *E.coli*, H_2_O_2_ degradation in the cell was severely hampered in the mutant strains compared to the wild-type (H_2_S proficient) strains [48]. H_2_S has the ability to antagonize H_2_O_2_ effects on the host [49], scavenge reactive oxygen species [50], and induce the expression of catalases and peroxidases [50], all leading to lower amounts of H_2_O_2_ in the host.

The ability of *Haemophilus* to utilize H_2_O_2_ could be one of the ways it may protect the host against reactivation. In addition to its aforementioned inverse correlation with viral load and its predictive potential in random forests, *Haemophilus*, like *Veillonella*, had a higher relative abundance in samples that came from subjects that never had a reactivation episode compared to samples that were negative for virus but that came from subjects that had a reactivation episode at least once (Figure S4B). In a study examining production and utilization of H_2_O_2_ by salivary bacteria, three species from the *Streptococcus mitis* group produced high levels of H_2_O_2_ when grown aerobically (2–9.8 mM) and anaerobically (1.1- 3.9 mM) which was rapidly cleared (within minutes) by *Haemophilus parainfluenzae* and *H. segnis* (as well as *Neisseria sicca* and *Staphylococcus epidermidis*). When these pure cultures were incubated with as high as 295 mM of H_2_O_2_, it was completely catabolized within 15 min [51].

There are also bacteria that may play a role in promoting viral reactivation and/or propagation. Strong positive correlations were found between EBV viral load and *Gracilibacteria* and *Abiotrophia* relative abundances, with *Gracilibacteria* being the most predictive taxa for whether a sample was positive or negative for virus. *Gracilibacteria* (a.k.a. GN02) is a candidate phylum, meaning that no cultured representatives have been found but it is known to exist through 16S rRNA amplicon or shotgun metagenomic sequencing analyses. It was first described in a study of the Guerrero Negro hypersaline microbial mat [52] and since then at least 13 clones have been detected from the human oral cavity [53]. In 2013, single cell genomics was performed on 2 clones from this candidate phylum and a re-coding of the opal stop codon UGA for glycine was discovered [54]. *Abiotrophia*, on the other hand, is more dominant in the oral cavity than *Gracilibacteria*, and one species, *A. defectiva*, is most known for its role in infective endocarditis [55]. There is no information available, as yet, on how these two taxa could promote viral reactivation and/or replication.

While points have been made as to how the bacteria that were predictive or correlative could play a role in preventing or promoting viral reactivation/replication, there is also the possibility that differences observed between the groups were a byproduct of viral reactivation and replication changing the microbiome. A future study with a larger sample size of astronauts that (i) never had viral reactivation, (ii) were always positive for viral titers, and (iii) at some time point experienced latency and at other points experiencing shedding, which could help answer the above question. A larger dataset would also help determine whether a signature microbiome that protects against or promotes reactivation/replication is different depending on whether the astronaut is on Earth or in space.

Based on the above data, the organisms that appear to play a role in viral reactivation or propagation and which in vitro studies may be useful, are *Gracilibacteria*, *Abiotrophia*, *Oribacterium*, *Veillonella*, and *Haemophilus*.

### Impact of spaceflight on the salivary microbiome

This study examined how the salivary microbiome changes as a result of spaceflight. Alpha diversity (richness, Shannon’s diversity, phylogenetic diversity) changed during flight but beta diversity (differences between samples based on what is there and their abundances) did not. While there were no population level differences as a result of spaceflight, 5 out of the 10 subjects did have distinct microbial communities pre-flight, in-flight, and post-flight. It is interesting to note that for the five subjects that had a significant difference in their microbiome, this was their first space mission, while the five subjects that did not have microbiome differences, had previously flown to the ISS. This may suggest that a person’s microbiome is able to adapt to spaceflight and is less recalcitrant to microbiome effects during spaceflight upon re-exposure. For those five subjects whose microbiome was not impacted by flight, it was evident that their post-flight samples were never similar to the pre-flight samples, even after 6 months, suggesting that the act of flying to the ISS causes either an irreversible change in the oral microbiome or a change that takes longer than 6 months to rebound. Further studies would need to be done to examine the consequence of these changes on the long-term health of the astronaut. Since each subject had different microbes influencing the microbial communities, a focus on a personalized astronaut microbiome to maintain health and prevent disease would be necessary.

## Conclusions

This is the first study to look at how the salivary microbiome changes as a result of spaceflight and the search for bacterial biomarkers for viral reactivation. Further studies examining the role of specific organisms that were shown to be correlative and predictive in viral reactivation, a serious problem in astronauts during spaceflight, could lead to mitigation strategies to help prevent disease during both short and long duration space missions.

## Methods

### Saliva sample collection and DNA extraction

Saliva samples were collected from 10 male astronauts at various time points over the course of their mission. The duration of spaceflight ranged from 2–9 months with an average ISS mission being 4.4 months. Samples were collected (i) two times pre-flight, at 180 days (L-180) and 90 (L-90) days before launch, (ii) three times during flight, at early (1–2 months on ISS) and mid (2–4 months on ISS), and late (10 days before landing) time points and (iii) four times post-flight, on landing day (R + 0), 30 days (R + 30), 60 days (R + 60), and 180 days (R + 180) after return. These time points were adjusted (+/− 30 days) according to the convenience of crew members. The samples were collected by the astronauts themselves by placing a synthetic polymer swab (Salimetrics LLC, State College, PA) in one’s mouth and swirling it around for 2–3 min until saturation. The samples were stored at − 80 °C until ready to process. Once all samples from a given subject was collected, they were thawed and then centrifuged at ×1400*g* for 5 min to separate the fluid from the swab. The separated fluid was stored at − 80 °C until DNA extraction and the swab discarded.

### DNA isolation

The frozen fluid was allowed to thaw on ice and then vortexed and centrifuged at ×16,000g for 20 min to make a cell pellet at the bottom of the tube. The supernatant was discarded, and the cell pellet was re-suspended in 200 μL PBS. DNA was extracted from this re-suspended pellet with a QIA-Amp DNA kit (Qiagen; Germantown, MD). DNA concentration was determined with the NanoDrop ND-1000 spectrophotometer (NanoDrop Technologies, Inc. Wilmington, DE).

### Detection and quantification of EBV, HSV-1, and VZV

Quantitative real-time PCR was performed in a TaqMan 7900 sequence detector (Applied Biosystems) using fluorescence-based amplification. Two microliters of viral DNA was added to 2× TaqMan^TM^ Fast Universal PCR master mix (Thermofischer, USA), with primers added to a final concentration of 100 nmol and probes added to a final concentration of 50 nmol. Primer sequences and probes for the herpes viruses (EBV, VZV, and HSV-1) along with the glyceraldehyde 6-phosphate dehydrogenase (GAPDH) DNA sequences are listed in Table 1 and have been described previously [22]. Viral DNA standards generated from each herpes virus ranging from 10^0^ to 10^6^ copies/μl were included in all reactions. Reactions were performed in triplicate and were as follows: initial denaturation at 94 °C for 20 s, followed by 40 cycles of 95 °C for 1 s, and 60 °C for 20 s. A sample was considered negative for virus if it had lower than 10 copies of viral DNA.

**Table 1:** Primer and probe sequences used for the viral load estimation

Name	Sequence
VZV (gene 63)	5′-CGCGTTTTGTACTCCGGG-3′ (forward) 5'-ACGGTTGATGTCCTCAACGAG-3′ (reverse) 5'-TGGGAGATCCACCCGGCCAG-3′ (probe)
VZV (gene 21)	5′-TGTTGGCATTGCCGTTGA-3′ (forward) 5′-ATAGAAGGACGGTCAGGAACCA-3′ (reverse) 5′-CTGCTTCCCCAGCACGTCCGTC-3′ (probe)
EBV	5′-CGGAAGCCCTCTGGACTTC-3′ (forward) 5′-CCCTGTTTATCCGATGGAATG-3′ (reverse) 5′-TGTACACGCACGAGAAATGCGCC-3′ (probe)
HSV1	5′-TGGTATTGCCCAACACTTTCC-3′ (forward) 5′-GCGCCAGGCACACACAT-3′ (reverse) 5-/FAM/CGTGTCGCGTGTGGT/BHQ_1/-3′ (probe)

### Microbiome analysis

The scripts used to perform the following processing and analyses are presented in Dataset S3.

### Library preparation and sequencing

The V3/V4 region (~ 460 bp) of the isolated genomic DNA was paired-end sequenced (2 × 300 bp) on the Illumina Mi-Seq platform following the protocol outlined by Illumina (https://support.illumina.com/documents/documentation/chemistry_documentation/16s/16s-metagenomic-library-prep-guide-15044223-b.pdf). To summarize, amplicon PCR was performed by adding 2.5ul of 5 ng/ul of DNA to 5 ul of 1uM Forward primer, 5ul of 1uM reverse primer, and 12.5 ul of 2× KAPA HiFi HotStart ReadyMix. Forward primer sequences are 5′TCGTCGGCAGCGTCAGATGTGTATAAGAGACAGCCTACGGGNGGCWGCAG 3′. Reverse primer sequences are 5′GTCTCGTGGGCTCGGAGATGGTATAAGAGACAGGACTACHVGGGTATCTAATCC 3′. The PCR condition were as follows: 95 °C for 3 min followed by 25 cycles of 95 °C for 30 s, 55 °C for 30s, and 72 °C for 30s. A final elongation step was performed at 72 °C for 5 min.

Following quality control and PCR clean up, index PCR was set up with the Nextera XT index kit and a limited-cycle amplification (8 cycles) to attach dual indices and adapters to the amplicons. The library was quantified using Qubit and normalized to 4 nM for all samples, after which all samples were pooled together. The pooled library was loaded onto the sequencer at 6pM with 10% phiX spiked in. The Miseq run utilized the v3 chemistry and 2 × 300 cycle sequencing kit (MS-102-3003). Samples were demultiplexed and adaptors removed using bcl2fastq from Illumina.

### 16S rRNA raw data processing and quality control

The DADA2 pipeline [56] (https://benjjneb.github.io/dada2/index.html) was used for quality control and processing (including chimera removal) of the raw demultiplexed reads resulting in an amplicon sequence variant (ASV) table, a “higher resolution analogue of the traditional OTU table.” The filter and trim parameters were truncLen = c (280, 220), trimLeft = c (17, 21), maxN = c (0, 0), maxEE = c (2, 2), truncQ = c (2, 2), and rm.phix = c (TRUE, TRUE).

Taxonomy was assigned with the SILVA reference database (version 128) (https://benjjneb.github.io/dada2/training.html) using both the “nr” and “species assignment” databases. A custom script was used to filter the ASV table to keep only those samples that had greater than 100 reads and to keep only those ASV that were at least 0.01% abundant in a sample. The NTC sequencing control had 170 reads, with none of the ASV shared between the samples. The two kit controls had 6094 and 2969 reads. ASVs that were found exclusively in the kit control were removed from the table, along with 14 ASVs that were found in the kit controls and at least one sample. The average number of reads across all samples was 126,694 (ranging from 6216 to 312,133) totaling 697 ASVs.

The positive control that was processed with DADA2 (92,514 reads) validated our pipeline as the sequence was only present in the positive control and had a 100% match with 100% coverage to the reference sequence.

### Bioinformatics analyses

Alpha diversity metrics (Shannon’s diversity, Richness, Phylogenetic diversity) were calculated in QIIME [57]. Multiple rarefactions were performed on the ASV table, with the following arguments: − m 1000 (seq/sample), − x 100000 (seq/sample), and − s 5000 (steps between min/max of seq/sample), with the number of reps being two, − n 2. For alpha diversity of *Streptococcus* ASVs, the conditions were as followed: − m 1000, − x 50000, − s 4000, and − n 1. The data generated in QIIME was plotted and visualized in Prism (v.7). Significance between pre-flight, flight, and post-flight samples was measured using the Kruskal-Wallis one-way analysis of variance, paired design, followed by the Benjamini-Hochberg multiple comparison post-hoc test in Prism. Linear regression analyses were also performed which produced the same results as the Kruskal-Wallis test. Significance was defined as *P* < 0.05.

Phyla and genus level data used in various analyses were summarized from ASV data using QIIME. NMDS, heatmaps, and boxplots were generated in R (http://www.R-project.org/) using centered log ratio (clr) transformed data (transformed using the “compositions” package in R) with a uniform prior of 0.5 added to each value before transformation [58, 59]. The ALDEx2 package in R was used to compare the relative abundances of phyla and genera between the different flights. Significance was based on the Benjamini-Hochberg corrected *p* value of the Wilcoxon rank test (significance threshold *P* < 0.05).

Spearman’s rho values and corresponding *p* values for correlation analyses between phyla (clr transformed data) from samples either positive or negative for virus (Fig. 5 data) were generated in R using the rcorr function. The p.adjust function was used to correct the *p* values using the Benjamini-Hochberg method. The heatmaps of rho values were generated using the heatmap.2 function in the “gplots” package.

Kendall tau and corresponding *p* values for correlation analysis between (i) Shannon’s diversity and qPCR viral load and (ii) observed richness and qPCR viral load was performed with the cor.test function in R for each rarefaction, followed by a false discovery rate *p* value correction using the p.adjust function.

Correlation analysis between the microbiome and viral load was performed using the ALDEx2 package as previously described [33]. Briefly, the Spearman’s rank correlation between the relative abundances of each genus (clr transformed) in 128 inferred technical replicates and the EBV and HSV-1 viral load as measured by qPCR was calculated using the aldex.corr function. Spearman’s rho values were converted to *p* values and corrected by the Benjamini-Hochberg procedure using the cor.test function in R. The heatmap of Spearman’s rho values was generated using the heatmap.2 function in the “gplots” package.

Random forests analysis was performed in R using the “randomForest,” “plyr,” “rfUtilities,” and “caret” packages on clr transformed data. LOOCV and OOB methods were employed using 500 decision trees, 1000 permutations, and 7 features to be randomly sampled at each node in the tree (i.e., mtry = 7).

## Supplementary information

**Additional file 1: Figure S1.** Alpha diversity of *Streptococcus* sequences in the saliva. Saliva samples were collected from 10 astronauts at various timepoints before flying to the ISS (pre-flight), while on the ISS (flight) and upon return to Earth (post-flight). Streptococcal diversity was measured using Shannon’s diversity index (A) and richness was measured by counting the number of unique *Staphylococcus* amplicon sequence variants within each sample (B) Reads were rarified to different counts (x- axis) and the values for each rarefied read count plotted (y-axis). Shannon’s diversity was lowest during flight compared to pre-flight and post-flight samples. Richness, on the other hand, was lowest during post-flight, with pre-flight and in-flight having similar number of observed *Streptococcus* sequences. **Figure S2.** Heatmap of read counts of genera detected in the saliva. ASVs were summarized, based on taxonomy, to the genus level and all those that could be assigned to a genus were included in this heatmap. Multiple samples were collected from each astronaut but were averaged for a given flight status. The data was then clr transformed. Clr values that are positive are higher than the geometric mean (and thus can be considered more abundant) and those that are negative are lower than the geometric mean (and can be considered less abundant). The heatmap is separated by subject and by flight status (i.e. pre-flight, in-flight or post-flight samples). In the heatmap, red represents the highest clr value and light green the lowest. **Figure S3.***Streptococcus* strains can predict viral status. Results from the random forest classifier showing the *Streptococcus* sequences that are the most discriminatory between viral positive (qPCR detection of EBV or HSC-1) and viral negative samples, in descending order. OOB error rate was 19% and LOOCV accuracy was 81%. **Figure S4.** Microbiome profiles in subjects with a history of viral reactivation vs those that never had viral shedding. (A) & (B) Boxplots of *Veillonella* and *Haemophilus* showing the relative abundances of each genus in (i) samples that were negative for virus but from subjects with a history of viral shedding (“history”) and (ii) samples from subjects that never had a reactivation episode “never”. These taxa were statistically significantly different (*p* < 0.05) between the groups. (C) Results from the random forest classifier showing the bacterial genera that are the most discriminatory between the “never” and “history” groups, in descending order. OOB error rate was 9% and LOOCV accuracy was 91%. *Veillonella* was the most predictive.

**Additional file 2.** Database S1.

**Additional file 3.** Database S2.

**Additional file 4.** Database S3.

## Data Availability

The raw Illumina sequencing reads have been submitted to NCBI’s short read archive (SRA) under accession number PRJNA539937. The data can also be found in NASA's GeneLab data repository under the following link:https://genelab-data.ndc.nasa.gov/genelab/accession/GLDS-280
