# Supplementary material for: The influence of spaceflight on the astronaut salivary microbiome and the search for a microbiome biomarker for viral reactivation
Source: Microbiome. 2020 Apr 20;8:56. doi: 10.1186/s40168-020-00830-z (PMC7171750; doi:10.1186/s40168-020-00830-z)
Supplement: Supplementary file 2 — Additional file 1: Figure S1. Alpha diversity of Streptococcus sequences in the saliva. Saliva samples were collected from 10 astronauts at various timepoints before flying to the ISS (pre-flight), while on the ISS (flight) and upon return to Earth (post-flight). Streptococcal diversity was measured using Shannon’s diversity index (A) and richness was measured by counting the number of unique Staphylococcus amplicon sequence variants within each sample (B) Reads were rarified to different counts (x- axis) and the values for each rarefied read count plotted (y-axis). Shannon’s diversity was lowest during flight compared to pre-flight and post-flight samples. Richness, on the other hand, was lowest during post-flight, with pre-flight and in-flight having similar number of observed Streptococcus sequences. Figure S2. Heatmap of read counts of genera detected in the saliva. ASVs were summarized, based on taxonomy, to the genus level and all those that could be assigned to a genus were included in this heatmap. Multiple samples were collected from each astronaut but were averaged for a given flight status. The data was then clr transformed. Clr values that are positive are higher than the geometric mean (and thus can be considered more abundant) and those that are negative are lower than the geometric mean (and can be considered less abundant). The heatmap is separated by subject and by flight status (i.e. pre-flight, in-flight or post-flight samples). In the heatmap, red represents the highest clr value and light green the lowest. Figure S3.Streptococcus strains can predict viral status. Results from the random forest classifier showing the Streptococcus sequences that are the most discriminatory between viral positive (qPCR detection of EBV or HSC-1) and viral negative samples, in descending order. OOB error rate was 19% and LOOCV accuracy was 81%. Figure S4. Microbiome profiles in subjects with a history of viral reactivation vs those that never had viral shedding. (A [file 40168_2020_830_MOESM1_ESM.pdf]

A

Figure S1

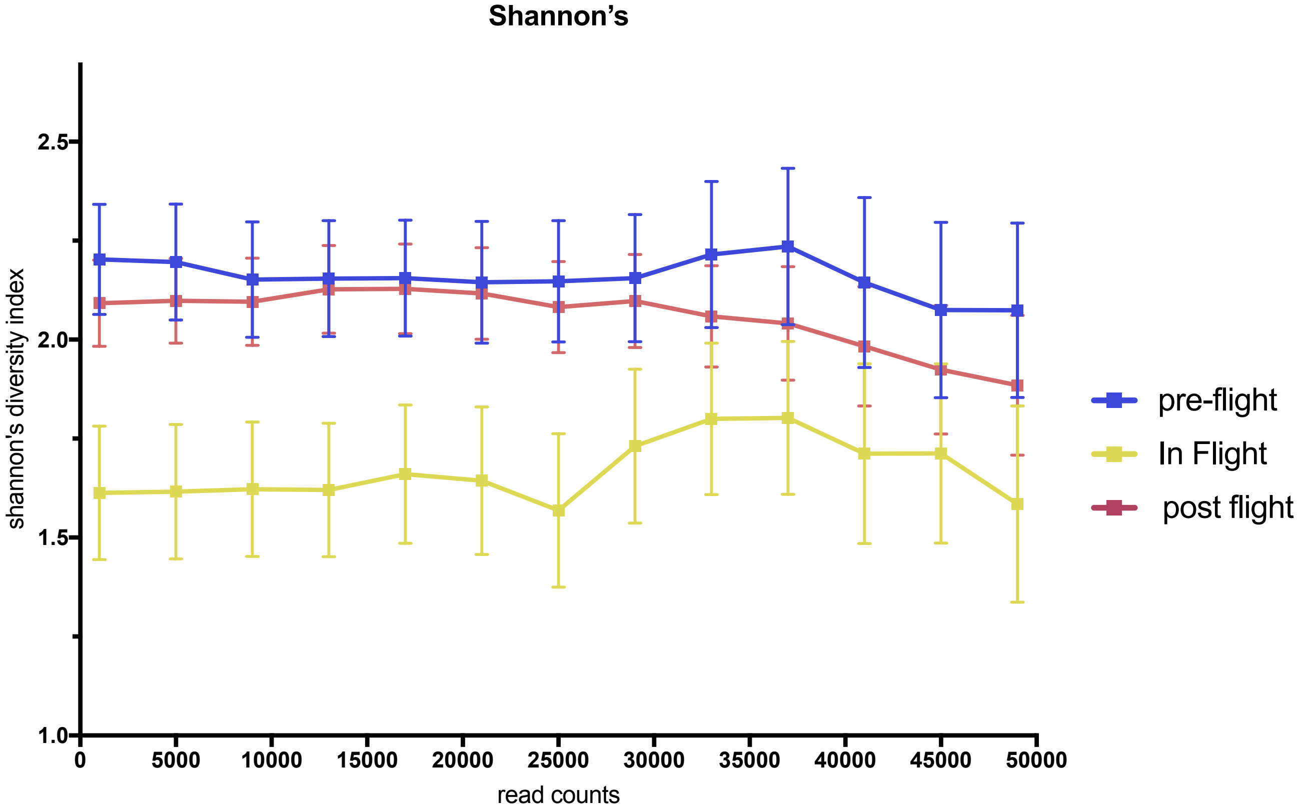

Shannon's
shannon's diversity index
2.5
2.0
1.5
1.0
0
5000
10000
15000
20000
25000
30000
35000
40000
45000
50000
read counts
pre-flight
In Flight
post flight

B

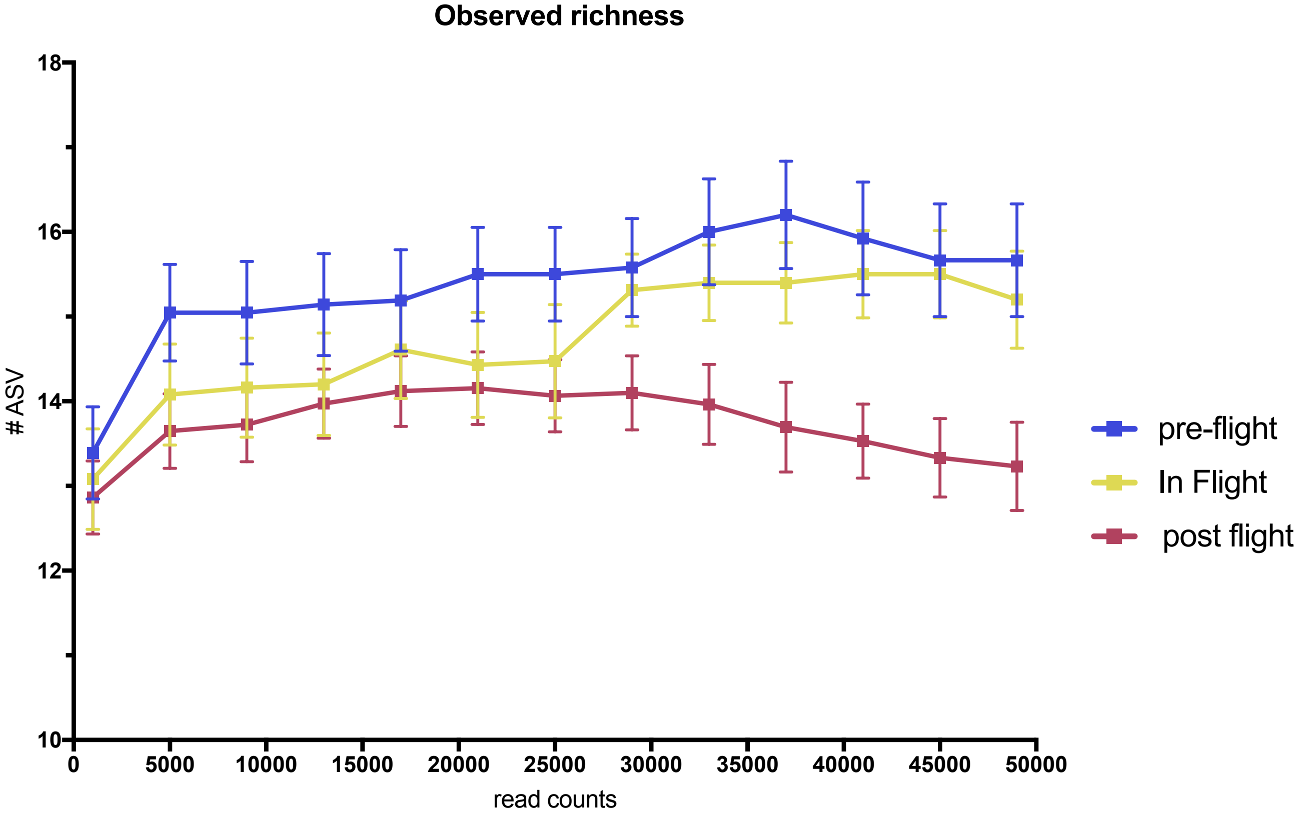

Observed richness
ASV
18
16
14
12
10
0
5000
10000
15000
20000
25000
30000
35000
40000
45000
50000
read counts
pre-flight
In Flight
post flight

Figure S2

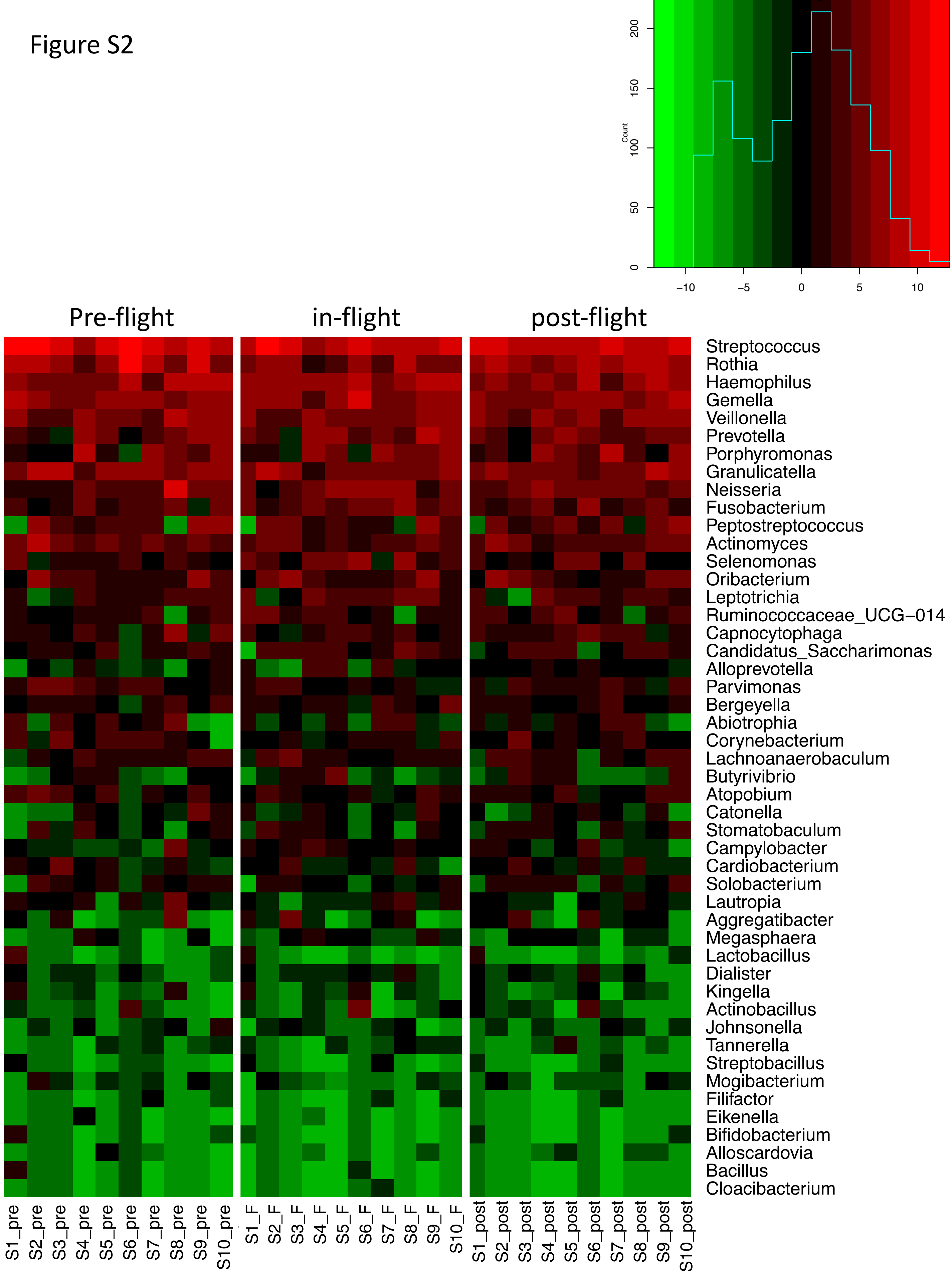
Count
0
50
100
150
200
−10
−5
0
5
10
Pre-flight
in-flight
post-flight
Streptococcus
Rothia
Haemophilus
Gemella
Veillonella
Prevotella
Porphyromonas
Granulicatella
Neisseria
Fusobacterium
Peptostreptococcus
Actinomyces
Selenomonas
Oribacterium
Leptotrichia
Ruminococcaceae_UCG−014
Capnocytophaga
Candidatus_Saccharimonas
Alloprevotella
Parvimonas
Bergeyella
Abiotrophia
Corynebacterium
Lachnoanaerobaculum
Butyrivibrio
Atopobium
Catonella
Stomatobaculum
Campylobacter
Cardiobacterium
Solobacterium
Lautropia
Aggregatibacter
Megasphaera
Lactobacillus
Dialister
Kingella
Actinobacillus
Johnsonella
Tannerella
Streptobacillus
Mogibacterium
Filifactor
Eikenella
Bifidobacterium
Alloscardovia
Bacillus
Cloacibacterium
S1_pre
S2_pre
S3_pre
S4_pre
S5_pre
S6_pre
S7_pre
S8_pre
S9_pre
S10_pre
S1_F
S2_F
S3_F
S4_F
S5_F
S6_F
S7_F
S8_F
S9_F
S10_F
S1_post
S2_post
S3_post
S4_post
S5_post
S6_post
S7_post
S8_post
S9_post
S10_post

Figure S3

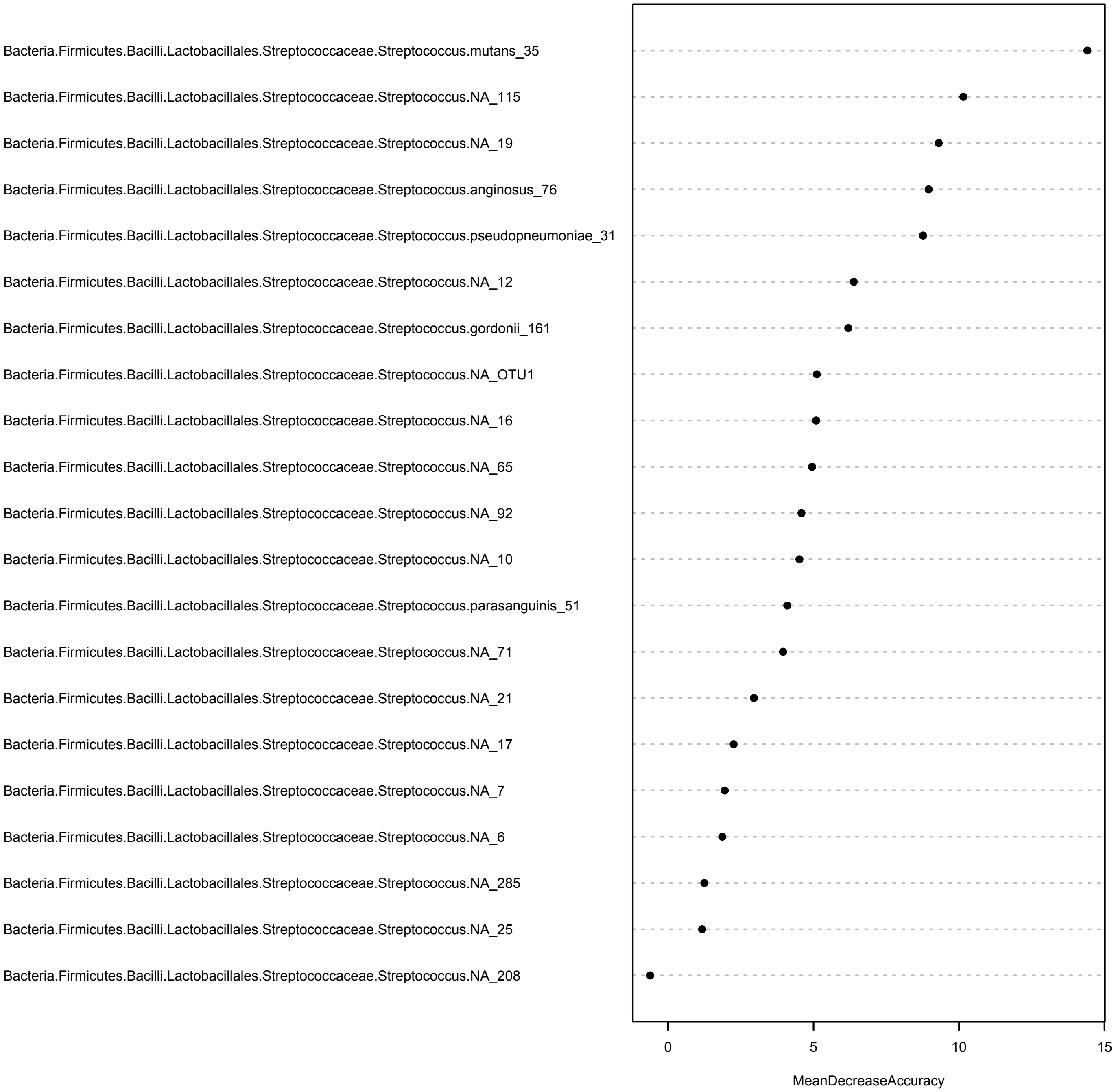

Bacteria.Firmicutes.Bacilli.Lactobacillales.Streptococcaceae.Streptococcus.mutans_35
Bacteria.Firmicutes.Bacilli.Lactobacillales.Streptococcaceae.Streptococcus.NA_115
Bacteria.Firmicutes.Bacilli.Lactobacillales.Streptococcaceae.Streptococcus.NA_19
Bacteria.Firmicutes.Bacilli.Lactobacillales.Streptococcaceae.Streptococcus.anginosus_76
Bacteria.Firmicutes.Bacilli.Lactobacillales.Streptococcaceae.Streptococcus.pseudopneumoniae_31
Bacteria.Firmicutes.Bacilli.Lactobacillales.Streptococcaceae.Streptococcus.NA_12
Bacteria.Firmicutes.Bacilli.Lactobacillales.Streptococcaceae.Streptococcus.gordonii_161
Bacteria.Firmicutes.Bacilli.Lactobacillales.Streptococcaceae.Streptococcus.NA_OTU1
Bacteria.Firmicutes.Bacilli.Lactobacillales.Streptococcaceae.Streptococcus.NA_16
Bacteria.Firmicutes.Bacilli.Lactobacillales.Streptococcaceae.Streptococcus.NA_65
Bacteria.Firmicutes.Bacilli.Lactobacillales.Streptococcaceae.Streptococcus.NA_92
Bacteria.Firmicutes.Bacilli.Lactobacillales.Streptococcaceae.Streptococcus.NA_10
Bacteria.Firmicutes.Bacilli.Lactobacillales.Streptococcaceae.Streptococcus.parasanguinis_51
Bacteria.Firmicutes.Bacilli.Lactobacillales.Streptococcaceae.Streptococcus.NA_71
Bacteria.Firmicutes.Bacilli.Lactobacillales.Streptococcaceae.Streptococcus.NA_21
Bacteria.Firmicutes.Bacilli.Lactobacillales.Streptococcaceae.Streptococcus.NA_17
Bacteria.Firmicutes.Bacilli.Lactobacillales.Streptococcaceae.Streptococcus.NA_7
Bacteria.Firmicutes.Bacilli.Lactobacillales.Streptococcaceae.Streptococcus.NA_6
Bacteria.Firmicutes.Bacilli.Lactobacillales.Streptococcaceae.Streptococcus.NA_285
Bacteria.Firmicutes.Bacilli.Lactobacillales.Streptococcaceae.Streptococcus.NA_25
Bacteria.Firmicutes.Bacilli.Lactobacillales.Streptococcaceae.Streptococcus.NA_208
0
5
10
15
MeanDecreaseAccuracy

Figure S4

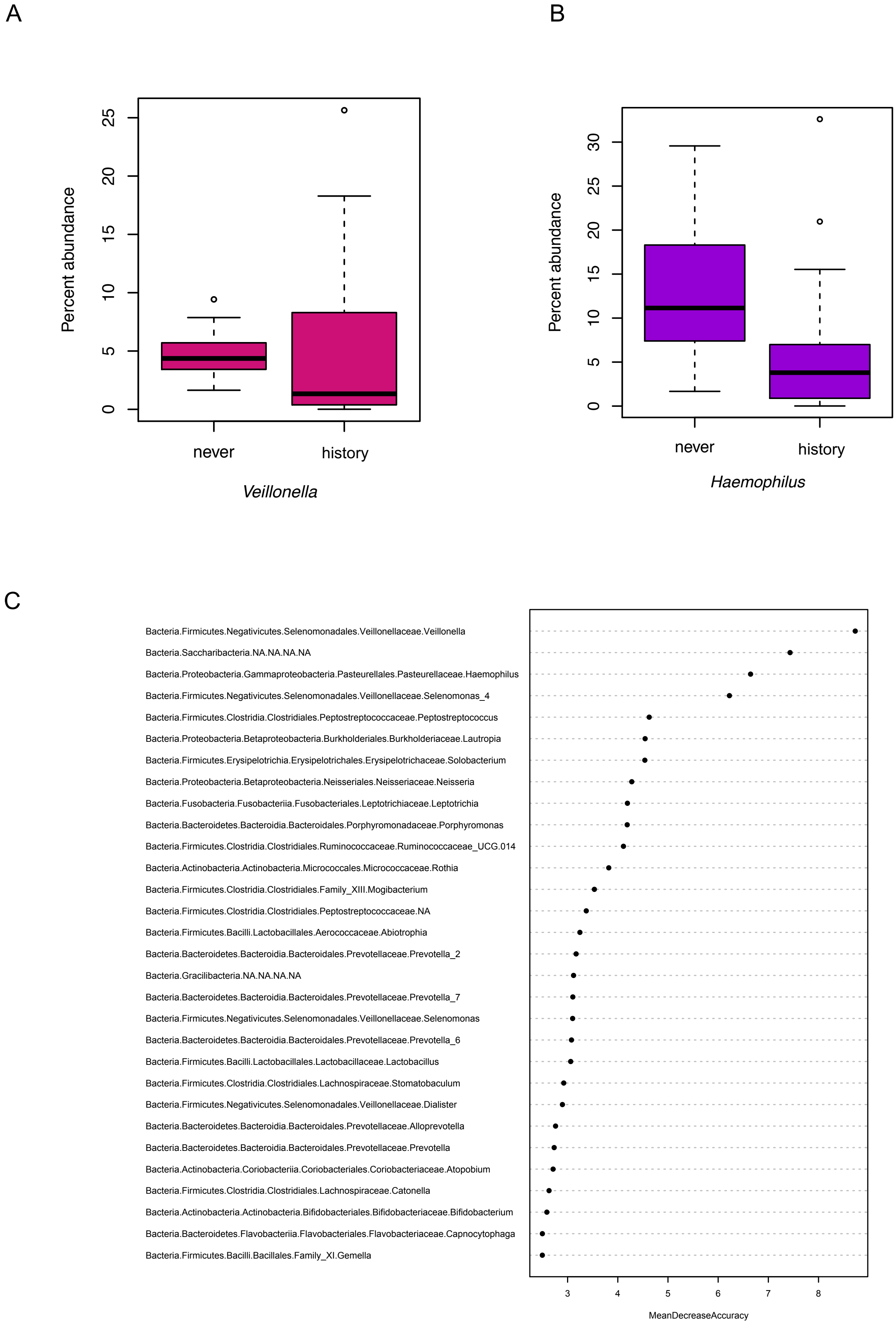

A
Percent abundance
0
5
10
15
20
25
never
history
Veillonella
B
Percent abundance
0
5
10
15
20
25
30
never
history
Haemophilus
C
Bacteria.Firmicutes.Negativicutes.Selenomonadales.Veillonellaceae.Veillonella
Bacteria.Saccharibacteria.NA.NA.NA.NA
Bacteria.Proteobacteria.Gammaproteobacteria.Pasteurellales.Pasteurellaceae.Haemophilus
Bacteria.Firmicutes.Negativicutes.Selenomonadales.Veillonellaceae.Selenomonas_4
Bacteria.Firmicutes.Clostridia.Clostridiales.Peptostreptococcaceae.Peptostreptococcus
Bacteria.Proteobacteria.Betaproteobacteria.Burkholderiales.Burkholderiaceae.Lautropia
Bacteria.Firmicutes.Erysipelotrichia.Erysipelotrichales.Erysipelotrichaceae.Solobacterium
Bacteria.Proteobacteria.Betaproteobacteria.Neisseriales.Neisseriaceae.Neisseria
Bacteria.Fusobacteria.Fusobacteriia.Fusobacteriales.Leptotrichiaceae.Leptotrichia
Bacteria.Bacteroidetes.Bacteroidia.Bacteroidales.Porphyromonadaceae.Porphyromonas
Bacteria.Firmicutes.Clostridia.Clostridiales.Ruminococcaceae.Ruminococcaceae_UCG.014
Bacteria.Actinobacteria.Actinobacteria.Micrococcales.Micrococcaceae.Rothia
Bacteria.Firmicutes.Clostridia.Clostridiales.Family_XIII.Mogibacterium
Bacteria.Firmicutes.Clostridia.Clostridiales.Peptostreptococcaceae.NA
Bacteria.Firmicutes.Bacilli.Lactobacillales.Aerococcaceae.Abiotrophia
Bacteria.Bacteroidetes.Bacteroidia.Bacteroidales.Prevotellaceae.Prevotella_2
Bacteria.Gracilibacteria.NA.NA.NA.NA
Bacteria.Bacteroidetes.Bacteroidia.Bacteroidales.Prevotellaceae.Prevotella_7
Bacteria.Firmicutes.Negativicutes.Selenomonadales.Veillonellaceae.Selenomonas
Bacteria.Bacteroidetes.Bacteroidia.Bacteroidales.Prevotellaceae.Prevotella_6
Bacteria.Firmicutes.Bacilli.Lactobacillales.Lactobacillaceae.Lactobacillus
Bacteria.Firmicutes.Clostridia.Clostridiales.Lachnospiraceae.Stomatobaculum
Bacteria.Firmicutes.Negativicutes.Selenomonadales.Veillonellaceae.Dialister
Bacteria.Bacteroidetes.Bacteroidia.Bacteroidales.Prevotellaceae.Alloprevotella
Bacteria.Bacteroidetes.Bacteroidia.Bacteroidales.Prevotellaceae.Prevotella
Bacteria.Actinobacteria.Coriobacteriia.Coriobacteriales.Coriobacteriaceae.Atopobium
Bacteria.Firmicutes.Clostridia.Clostridiales.Lachnospiraceae.Catonella
Bacteria.Actinobacteria.Actinobacteria.Bifidobacteriales.Bifidobacteriaceae.Bifidobacterium
Bacteria.Bacteroidetes.Flavobacteriia.Flavobacteriales.Flavobacteriaceae.Capnocytophaga
Bacteria.Firmicutes.Bacilli.Bacillales.Family_XI.Gemella
3
4
5
6
7
8
MeanDecreaseAccuracy
